# Extended-Spectrum β-Lactamase–producing *Enterobacteriaceae* among Travelers from the Netherlands

**DOI:** 10.3201/eid.1908.130257

**Published:** 2013-08

**Authors:** Sunita Paltansing, Jessica A. Vlot, Margriet E.M. Kraakman, Romy Mesman, Marguerite L. Bruijning, Alexandra T. Bernards, Leo G. Visser, Karin Ellen Veldkamp

**Affiliations:** Leiden University Medical Center, Leiden, the Netherlands

**Keywords:** *Enterobacteriaceae*, *Escherichia coli*, *Klebsiella pneumoniae*, extended-spectrum β-lactamase, bacteria, antibiotic, sequence type, Holland, the Netherlands, antimicrobial resistance, ESBL, ESBL-E, carbapenemase, CP-E, travel health, travelers

## Abstract

A prospective cohort study was performed among travelers from the Netherlands to investigate the acquisition of carbapenemase-producing *Enterobacteriaceae* (CP-E) and extended-spectrum β-lactamase–producing *Enterobacteriaceae* (ESBL-E) and associated risk factors. Questionnaires were administered and rectal swab samples were collected and tested before and after traveler return. Of 370 travelers, 32 (8.6%) were colonized with ESBL-E before trave,; 113 (30.5%) acquired an ESBL-E during travel, and 26 were still colonized 6 months after return. No CP-E were found. Independent risk factors for ESBL-E acquisition were travel to South and East Asia. Multilocus sequence typing showed extensive genetic diversity among *Escherichia coli*. Predominant ESBLs were CTX-M enzymes. The acquisition rate, 30.5%, of ESBL-E in travelers from the Netherlands to all destinations studied was high. Active surveillance for ESBL-E and CP-E and contact isolation precautions may be recommended at admission to medical facilities for patients who traveled to Asia during the previous 6 months.

The effect of international travel on the spread of multidrug-resistant *Enterobacteriaceae* (MDR-E) became more evident during 2007–2010. Data obtained during that time from prospective studies of returning travelers from Australia, Canada, Sweden, and the United States (New York, New York) revealed high rates of extended-spectrum β-lactamase producing *Enterobacteriaceae* (ESBL-E) carriage, varying from 18% to 25% after foreign travel (*1**–**4*). Two of these studies also reported a pretravel ESBL-E carriage rate of 7.8%.

The identification of carbapenemase-producing *Enterobacteriaceae* (CP-E) produced another set of challenges. Carbapenemases, such as *Klebsiella pneumoniae* carbapenemases (KPC), New Delhi metallo-β-lactamase (NDM), OXA-48, VIM and IMP, are plasmid-encoded enzymes, which have emerged worldwide. The rate of acquisition of CP-E during foreign travel is unknown; no surveillance system to date tracks these rates, and such rates are included sporadically in case reports, such as the situation recently reviewed by Van der Bij and Pitout (*5*). In the Netherlands, CP-E were found for the first time in 2010 (*6*).

No data were available on the pre-and post-travel carriage rates among travelers from the Netherlands. Our objective was to investigate whether these travelers are at risk of MDR-E (ESBL-E and/or CP-E) by use of a prospective cohort study design. Because detailed microbiological data of the isolates and epidemiologic data are crucial for assessing the real public health impacts of these organisms, we also investigated the persistence of intestinal colonization and possible spread to household contacts 6 months after the travelers returned.

## Materials and Methods

### Study Design

A prospective cohort study was conducted at the travel clinic at the Leiden University Medical Center and at the Hollands Midden Municipal Health Services in Leiden, the Netherlands. During March–September 2011, all adults who made an appointment for travel advice and had the intention to travel to areas outside Europe, North America, and Australia were invited to participate in the study. Travelers <18 years of age and those who traveled >3 months were excluded. Only 1 person in a couple or travel group was included.

Participants were asked to complete an electronic questionnaire and to deliver a rectal swab sample immediately before and immediately after travel. Questionnaires were used to collect demographic data, previous medical history, and travel information. Travelers who acquired MDR-E after foreign travel were asked to fill out a third questionnaire and deliver a third rectal swab 6 months after return.

If travelers were positive for MDR-E 6 months after return, their household contacts were also requested to submit a rectal swab and questionnaire. Household contacts were defined as persons who shared the same household with a participant on a regular basis. MDR-E–positive participants were asked to deliver a fourth rectal swab at the same time. The study was approved by the Leiden University Medical Center medical ethics committee.

### Bacterial Isolates

Rectal swab samples were collected with Stuart Agar Gel Medium Transport Swabs (Copan Diagnostics, Corona, CA). The swabs were saturated in trypticase soy broth supplemented with cefotaxime 0.25 mg/L and vancomycin 8 mg/L (MP products, Groningen, the Netherlands) and incubated for 24 hours at 37°C. After overnight incubation, the trypticase soy broth samples were subcultured on chromogenic ESBL screening agar (ESBL-ID; bioMérieux, Marcy-l’Étoile, France) and sheep blood agar as a growth control. All gram-negative rods growing on the ESBL-ID were identified by using MaldiTof-MS with BioTyper software version 3.0 (Bruker Daltonics, Breman, Germany), and antimicrobial drug susceptibility testing was performed by using the VITEK2 system (BioMérieux). All isolates underwent ESBL confirmatory disk testing by disk diffusion for ceftazidime and cefotaxime or cefepime (in cefoxitin-resistant isolates), with and without clavulanic acid, as recommended by Clinical and Laboratory Standards Institute guidelines (www.clsi.org).

MICs for meropenem and ertapenem were determined by using Etests (AB Biodisk, Solna, Sweden) according to the manufacturer’s instructions. MICs were interpreted by using EUCAST criteria (www.eucast.org/clinical_breakpoints/).

### Molecular Characterization of β-Lactamases

Molecular characterization of the β-lactamase genes in ESBL-E was performed by using Check-MDR CT103 version 1.1 (Check-Points B.V., Wageningen, the Netherlands) to test microarrays. The principals of the microarray system and interpretation software have been described (*7*). Concisely, the system combines ligation-mediated amplification with the detection of amplified products on a microarray to detect the various carbapenemase genes: OXA-48, NDM-1, IMP, VIM, and KPC; CTX-M groups: CTX-M group 1, 2, 9 or combined 8/25; and the most prevalent ESBL-associated single-nucleotide polymorphisms in TEM and SHV-variants. Furthermore, the 6 plasmid-mediated AmpC β-lactamases can be identified (www.lahey.org/studies).

### Molecular Typing of *Escherichia coli* isolates

Multilocus sequence typing (MLST) was performed on all *E. coli* isolates by using 7 housekeeping genes (*adk, fumC, gyrB, icd, mdh, purA*, and *recA*) to determine the corresponding sequence type (ST) and to designate the sequence type complex (STC) by using the MLST Databases at the Environmental Research Institute, University College Cork website (http://mlst.ucc.ie/mlst/dbs/Ecoli).

### Data analysis

A logistic regression model was used to determine risk factors for the acquisition of ESBL-E/CP-E after foreign travel for a total of 338 participants. Associations between acquiring an ESBL-E/CP-E after travel and different variables are calculated as odds ratios and p-values. Participants who were positive for ESBL-E/CP-E before travel were analyzed separately. Database processing and statistical analyses (univariate and multivariate analysis) were performed by using the SPSS software version 20.0 (SPSS Inc., Chicago, IL, USA). MLST analysis was performed by using BioNumerics software v.6.6 (Applied Maths, St-Martens-Lathem, Belgium).

## Results

### Study Population and Travel Characteristics

**Figure 1:**
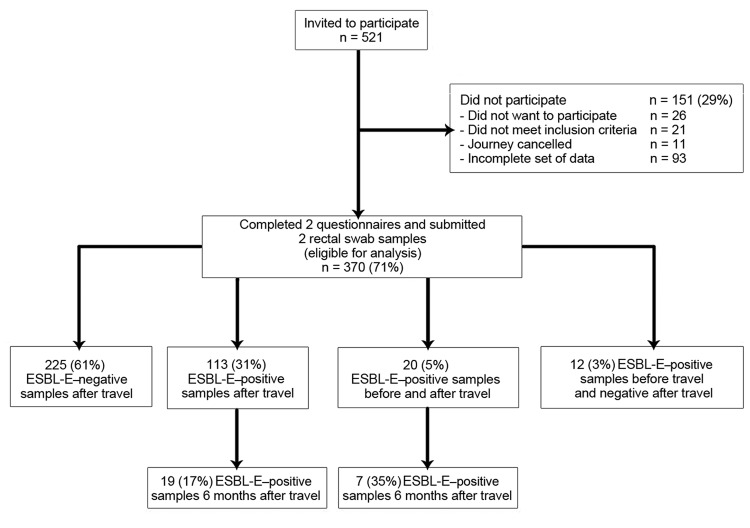
Participant colonization by *Enterobacteriaceae* species immediately before, immediately after, and 6 months after travel. ESBL-E, extended-spectrum β-lactamase producing–*Enterobacteriaceae.*

In total, 521 travelers were invited to participate in the study; 370 travelers completed 2 questionnaires and sent in 2 rectal swabs and were included in the analysis (Figure 1). The median age of the study population was 33 years (range 19–82), and 234 (63.2%) were women. The median length of stay abroad was 21 days (range 6–90 days). The most common reason for travel was vacation (n = 277).

Of the 370 participants, 113 (30.5%) whose pretravel swab samples were negative acquired MDR-E during foreign travel. Of these 113 participants, 19 (16.8%) still carried MDR-E 6 months after return. In 32 of the 370 participants (8.6%), MDR-E was identified before travel. Twenty (62.5%) of these 32 participants returned with MDR-E, 7 (35.0%) of whom were still colonized after 6 months. No MDR-E was found before or after travel in 225 (60.8%) participants.

### Travel-associated Risk Factors for ESBL Acquisition in Returning Travelers

For the analysis of travel-associated risk factors, data for 338 participating returning travelers with negative pretravel rectal swab sample test results were used (Technical Appendix Table 1). In total, 65 countries were visited; these are subdivided in 10 subcontinents. The most common destinations were Indonesia (n = 62), Thailand (n = 30), Malaysia (n = 27), Cambodia (n = 21), People’s Republic of China (n = 39), Kenya (n = 30), Tanzania (n = 24), Surinam (n = 20), and South Africa (n = 19).

The highest ESBL-E acquisition rates were identified among participants who visited countries in Asia: 73% in South Asia and 67% in East Asia. Univariate and multivariate analyses showed that the travel destinations South and East Asia were significant risk factors for the acquisition of ESBL-E (p<0.001). Participants traveling to Asia (all subcontinents) were more likely to return with ESBL-E colonization after a self-arranged trip (odds ratio 1.7; p = 0.07) or if they stayed in hostels/lodges (odds ratio 1.9; p = 0.08), although this finding was not statistically significant. There were no other risk factors for the acquisition of ESBL-E after foreign travel. The incidence proportions of ESBL-E after foreign travel are listed in Table 1.

**Table 1:** Incidence proportions and incidence rates for extended-spectrum β-lactamase producing *Enterobacteriaceae* colonization in 338 travelers from the Netherlands*

Destination	No. travelers	No. (%) travelers with ESBL-E after return	Incidence proportion, % (SE)	Person-days, all travelers	Mean duration of travel, all travelers, d	ESBL incidence rate/100 pdt (SE)
Southeast Asia	110	37 (34)	34 (4.5)	2,980	27	1.24 (0.20)
East Asia	33	22 (67)	67 (8.3)	776	24	2.83 (0.60)
South Asia	25	18 (72)	72 (9.2)	599	24	3.01 (0.70)
Central Asia	3	1 (30)	33 (33.3)	94	31	1.06 (1.06)
North Africa	10	4 (40)	40 (16.3)	112	11.2	3.57 (1.76)†
Central Africa	56	17 (30)	30 (6.2)	1,637	29	1.04 (0.25)
Southern Africa	26	3 (12)	12 (6.6)	631	25	0.48 (0.27)
Middle East	15	2 (13)	13 (9.1)	222	14.8	0.90 (0.64)
Central America and the Caribbean	28	7 (25)	25 (8.3)	544	19	1.29 (0.48)
South America	32	2 (6)	6 (4.4)	922	29	0.22 (0.15)
Total	338	113 (33)	33 (2.6)	8,536	25	1.32 (0.12)

*ESBL-E, extended-spectrum β-lactamase–producing *Enterobacteriaceae*; SE, standard error; pdt, person-days of travel. †The ESBL incidence rate/100 pdt is represented by 4 travelers returning from North Africa who carried ESBL-E: 3 of them had traveled for 7 days and 1 had a 25-day stay abroad, which accounts for the high SE.

### Microbiological Results and Molecular Characterization

A total of 133 participants were colonized with MDR-E after travel. This group consisted of 113 travelers who had initially negative pretravel swab samples. In addition, 20 participants who had positive pretravel samples also returned colonized with MDR-E. The ESBL-E of these 133 post-travel swab samples consisted of 146 *E. coli*, 10 *K. pneumoniae*, and 2 *Enterobacter cloacae* isolates.

No carbapenemase-producing MDR-E were found among the pre-and post-travel isolates. Molecular characterization of the post-travel isolates demonstrated that CTX-M group 1 ESBL (n = 110) predominated (CTX-M-1–like, n = 4; CTX-M-3–like, n = 1; CTX-M-15–like, n = 85; CTX-M-32–like, n = 20), followed by CTX-M group 9 ESBL (n = 42), CTX-M group 2 (n = 2), and CTX-M group8/25 (n = 1). One *E. coli* isolate carried an SHV-ESBL (238S+240K). In addition, some isolates coproduced plasmid-mediated AmpC β-lactamase, ACT/MIR (n = 1) or CMY-2 (n = 2).

Thirty-four ESBL-E were isolated from pretravel rectal swab samples from 32 participants: 29 (85.3%) samples were positive for *E. coli,* 4 for *K. pneumoniae* (11.8%), and 1 for *Citrobacter freundii* (2.9%). The CTX-M group 1 ESBL (n = 22) comprised (CTX-M–1 like, n = 4; CTX-M-15–like, n = 16; CTX-M-32–like, n = 2); the remaining ESBL isolates belonged to CTX-M group 9 ESBL (n = 8) and CTX-M group 2 (n = 1). Two *E. coli* isolates carried an SHV-ESBL (238S+240K).

Coresistance to other classes of antimicrobial drugs was common in pre- and post-travel isolates; 67% displayed resistance to trimethoprim/sulfamethoxazole, 36% to ciprofloxacin, 37% to tobramycin, 35% to gentamicin, and 29% to nitrofurantoin. All isolates were susceptible to colistin and carbapenems.

### MLST of ESBL-producing *E. coli* Isolates

**Figure 2:**
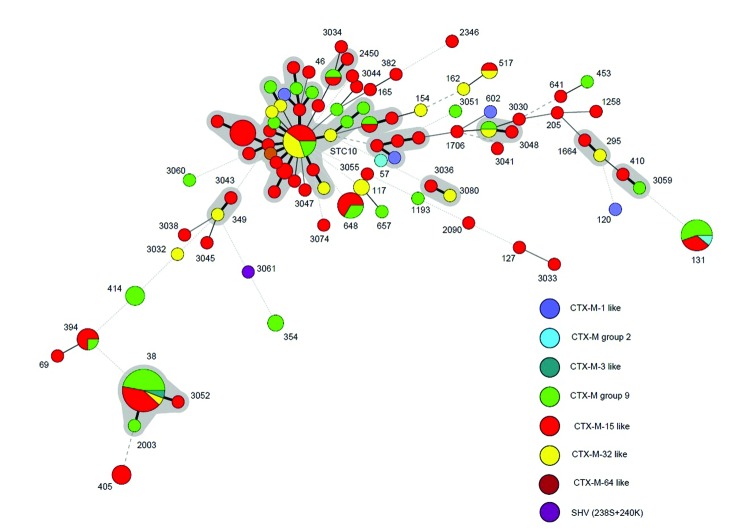
Multilocus sequence typing of *Escherichia coli* (n = 146) from the post-travel isolates of 133 travelers from the Netherlands. The numbers indicate the most prevalent sequence types (STs). Gray shadow indicates that >1 ST belongs to the same complex. The following sequences belong to STC10: ST4,10, 34, 43, 44, 48, 167, 193, 215, 218, 227, and 617. Thick connecting lines indicate single-locus variants; thin connecting lines indicate variants with 2–3 loci differences; dashed connecting lines indicate variants with 4 loci differences; dotted connecting lines indicate 5–7 loci differences.

MLST of 146 *E. coli* isolates from the post-travel samples identified 86 different STs; 31 new STs were found. The most prevalent STs were: ST38 (12%; n = 17), ST10 (7%; n = 10), and ST131 (4%; n = 9). The distribution of the CTX-M groups and types and STs is displayed in Figure 2. There was no association between ST and ESBL type, nor were STs associated with specific travel destinations. Pretravel isolates showed a similar diversity of STs, of which 3 were ST131. 

### Prolonged Carriage and Household Contacts

Of the 133 participants whose samples were positive for ESBL-E after return, 127 (95.4%) completed the follow-up survey and provided samples after 6 months. ESBL-E was isolated from 26 (20.4%) samples (Table 2). None of these participants reported the use of antimicrobial drugs or were hospitalized during the previous 6 months; none were health care workers, and none reported contact with farm animals. Diarrhea was reported by 7 participants. 

**Table 2:** Microbiological and molecular characteristics of rectal swab samples collected from travelers from the Netherlands immediately pre- and post–travel and 6 mo after return*

ID					Immediate post-travel samples								Post-travel sample 6 mo after return, isolate 3†		
	Pretravel sample				Isolate 1				Isolate 2						
	Species	CTX-M group	ST		Species	CTX-M group	ST		Species	CTX-M group	ST		Species	CTX-M group	ST
25	Neg	NA	NA		*E. coli*	9	131		None	NA	NA		*E. coli*	9	131
45	Neg	NA	NA		*E. coli*	1	405		*E. coli*	9	38		*E. coli*	1	405
56	Neg	NA	NA		*E. coli*	1	3036		*E. coli*	1	517		*E. coli*	1	3267
60	Neg	NA	NA		*E. coli*	1	648		*K.p.*	1	ND		*E. coli*	1	648
61	Neg	NA	NA		*E. coli*	1	648		*E. coli*	9	227		*E. coli*	1	131
62	Neg	NA	NA		*E. coli*	9	3037		None	NA	NA		*E. coli*	9	501
80	Neg	NA	NA		*E. coli*	1	131		None	NA	NA		*E. coli*	9	1177
86	Neg	NA	NA		*E. coli*	1	93		*E. coli*	1	2090		*E. cloacae*	9	ND
137	Neg	NA	NA		*E. coli*	1	155		*E. coli*	1	617		*E. coli*	9	131
204	Neg	NA	NA		*E. coli*	1	38		None	NA	NA		*K.p.*	1	ND
211	Neg	NA	NA		*E. coli*	1	3044		None	NA	NA		*K.p.*	1	ND
222	Neg	NA	NA		*E. coli*	9	2003		None	NA	NA		*E. coli*	9	2003
238	Neg	NA	NA		*E. coli*	9	414		None	NA	NA		*E. coli*	9	10
251	Neg	NA	NA		*E. coli*	1	34		None	NA	NA		*E. coli*	1	450
309	Neg	NA	NA		*E. coli*	1	3045		None	NA	NA		*E. coli*	1	3045
373	Neg	NA	NA		*E. coli*	1	38		None	NA	NA		*E. coli*	1	3266
387	Neg	NA	NA		*E. coli*	1	131		None	NA	NA		*E. coli*	1	131
454	Neg	NA	NA		*E. coli*	9	10		None	NA	NA		*E. coli*	9	10
474	Neg	NA	NA		*E. coli*	1	154		None	NA	NA		*E. coli*	1	131
12	*E. coli*	9	38		*E. coli*	1	3074		None	NA	NA		*E. coli*	1	38
105	*E. coli*	1	191		*E. coli*	1	120		*E. coli*	1	38		*E. coli*	1	120
255	*E. coli*	9	131		*E. coli*	1	617		None	NA	NA		*E. coli*	9	131
269	*K.p.*	1	ND		*K.p.*	1	ND		None	NA	NA		*K.p.*	1	ND
283	*E. coli*	9	131		*E. coli*	1	46		None	NA	NA		*E. coli*	9	131
505	*E. coli*	1	1163		*E. coli*	1	69		None	NA	NA		*E. coli*	9	3268
512	*E. coli*	9	657		*E. coli*	9	657		None	NA	NA		*E. coli*	1	657

*ID, participant identification number; CTX-M, extended-spectrum β-lactamase enzyme; ST, sequence type; Neg, no species were isolated from sample; *E.*, *Escherichia*; *K.p.*, *Klebsiella pneumoniae*; NA, not applicable; ND, no sequence type data available. †None of the participants with a positive rectal swab sample after 6 months reported antimicrobial drug use during the 6 months after return.

Of 113 participants who had initially negative pretravel samples and positive samples immediately after return, 19 (16.8%) were still colonized after 6 months. Of these, 7 participants had samples that were positive for *E. coli* with the same ST 6 months after return. Nine participants were positive for *E. coli* and had a different ST 6 months after return; 3 were positive for a different species 6 months after return. Eleven household contacts of 4 MDR-E–positive participants agreed to cooperate and submitted a rectal swab sample. ESBL-producing *E. coli* was isolated from 2 (18.1%) household contacts, each from different households. The first household contact carried a different ESBL-producing *E. coli* than the associated traveler before and after the trip. Both isolates carried a CTX-M group 9 enzyme. The second household contact was positive for SHV-ESBL-producing *E. coli* ST2599. The associated traveler’s samples were positive for *E. coli* ST617 and ST38 immediately after the trip, *K. pneumoniae* 6 months after return, and the fourth rectal swab sample was positive for a CTX-M-15–like *E. coli* ST3363.

Of 20 participants whose samples were positive before and after return, 7 (35.0%) participants were still colonized 6 months after return. Of these 7 participants, 5 carried a similar strain: 2 carried a CTX-M group 9–producing *E. coli* with an identical ST as before the trip, 2 carried a similar ST but with a different CTX-M group enzyme as before the trip, and 1 participant carried a CTX-M group 1–producing *K. pneumoniae* during the study period; 2 participants returned with *E. coli* with a different ST. No household contacts were included in this subgroup of travelers.

## Discussion

The results of this study show a high ESBL-E carriage rate of 30.5% among healthy participating travelers from the Netherlands after return. This finding is worrisome, because this ESBL-E carriage rate is higher compared with those in recent studies that identified international travel as an independent risk factor for ESBL-E colonization (*1**–**4*). It is striking that none of the potential travel-associated risk factors investigated in this study, other than traveling to South and East Asia, were found to contribute to this high ESBL-E carriage rate. Additional risk factors were not revealed by including in the univariate analysis the 13 participants who had a positive pretravel sample and acquired an ESBL-producing *E. coli* during travel with a different ST than before the trip. 

Tangden et al. associated gastroenteritis during travel with the risk for ESBL-E acquisition among travelers from Sweden (*3*). That association was not found in this study, which may reflect less fecal–oral contamination while traveling. Baaten et al. reported that diseases transmitted by the fecal–oral route among travelers to nonindustrialized countries have declined because of improved hygiene standards at the destination as measured by the human developmental index, sanitation index, and the water source index (*8*). The sanitation index levels, which represent the proportion of the population that has access to sanitation, were the lowest for sub-Saharan Africa and the Indian subcontinent. On the basis of these indices, we would expect the incidence of ESBL-E acquisition to be similar among travelers in countries in Asia and Africa. Nonetheless, participating travelers to Asia had the highest post-travel colonization rates. Travelers to Asia most likely differ in their eating habits compared with travelers to African countries, since the former are more likely to eat in individual establishments outside of hotels or from street vendors. Thus, the high incidence rate found for returning travelers from Asia in this study may result from the increased risk for foodborne exposure.

No CP-E were found despite the fact that countries were visited where CP-E are prevalent in hospitals and in the environment (*5*). Other known risk areas besides India for the acquisition of CP-E, such as the United States, Greece, Italy, and the Balkan region were not included in this study, because these travelers do not visit the Travel Clinic of the Leiden University Medical Center. Many citizens from the Netherlands have relatives in North African countries or Turkey whom they visit frequently. OXA-48–producing bacteria are endemic to these countries (*9*). These travelers do not consult travel clinics and may well return carrying OXA-48–producing isolates unnoticed.

Peirano et. al. (*2*) reported that the prevalence of ST131, a uropathogenic *E. coli* notorious for its worldwide expansion and spread of CTX-M-15, was similar among travelers and non-travelers from the Calgary region. The most prevalent ESBL among the travelers participating in this study was the CTX-M-15–like enzyme. However, this enzyme was found in a plethora of different STs of *E. coli*. Participants in the Leiden area not only showed a great heterogeneity of STs but also harbored different CTX-M types after travel and 6 months after return. The majority of the *E. coli* strains identified in the participants in this study were of STs that clustered around ST10 and belonged to ST complex 10 (STC10). STC10 strains essentially belong to the nonvirulent, commensal phylogenetic group A (*10*). In a recent study based in France, isolates belonging to STC10 were found to be the most prevalent among fecal samples from healthy carriers of nalidixic acid–resistant (but ESBL-negative) *E. coli* (*11*). It is also the most prevalent STC in the MLST database. Data from this study show that transmissible genetic elements containing resistance genes are exchanged with naive *E. coli* strains of the human intestinal microbiota during foreign travel combined with foodborne exposure.

Although 26 participants had positive results for ESBL-E 6 months after travel, they were not all positive for the same enterobacterial strain that was identified immediately after travel. In 8 participating travelers colonized with *E. coli*, an ESBL of the same CTX-M group was identified in the immediate post-travel sample as after 6 months, but *E. coli* with a different ST was detected. In 11 travelers, the strain persisted during the study period. It is possible that more strain types were present in the rectal samples where colony morphology of different strains was not discriminative. However, it is also possible that the transfer of ESBL genes between strains within a host is a frequent occurrence. Or, the acquisition of a new ESBL-E occurs at the expense of the resident strain.

Interhousehold transmission of ESBL-E has been demonstrated in the community setting (*12**,**13*). Clonally related strains could be found for 66% of the isolates from infected community patients and their corresponding household contacts (*13*). Because of the limited data on household contacts in the present study, the transmission dynamics of ESBL-E in households after foreign travel remain to be discovered.

The high pretravel ESBL-E carriage rate among our study participants (8.6%) was an unexpected finding. Two recent studies on the ESBL-E carriage rate in the community have been conducted in the Amsterdam area. In the first study, 10.1% of the fecal samples from outpatients with gastrointestinal discomfort being assessed by their general practitioners yielded ESBL-E, predominantly CTX-M-15–producing *E. coli* (*14*). In a second study, investigating the prevalence of ESBL-E carriage in the general community, a carriage rate of 8.5% was found (E.A. Reuland et al., unpub. data). Although no data on travel history were given, the investigators pointed out that foreign travel might be responsible for at least part of ESBL-E carriage rates among outpatients from the Netherlands. This finding is supported by data from our study: 50% of participants who had a positive pretravel sample had traveled during the previous 12 months. This high percentage of carriers identified in this study before travel points toward ongoing importation of ESBL-E. Other potential reservoirs for ESBL-E are poultry and other retail meats, which have been found to be contaminated with ESBL-producing *E. coli* strains harboring the genes on identical plasmids as found in human isolates (*15**,**16*).

International travel is growing and the number of intercontinental flights has increased during the past decade. The findings in this study support the role of international travel on the ESBL-E acquisition and carriage rates among travelers from the Netherlands, especially to South and East Asia. The high pre- and post–travel carriage rates among persons traveling from the Netherlands indicate that the consequences of increased foreign travel are already manifest in this country. The lack of apparent travel-associated risk factors, the spread of CTX-M enzymes through a highly diverse population of *E. coli,* the association of ESBL production with multidrug resistance, and the possible role of other sources make containing the spread difficult. These factors also complicate the implementation of other strategies, such as pretravel advice, and imply that all travelers to Asia should be considered for carriage of ESBL-E. Although CP-E were not found in this study, CP-E have been introduced into the Netherlands by returning travelers (*6**,**17**–**19*), and introduction by asymptomatic travelers to the Netherlands from countries where CP-E are endemic may largely go unnoticed. There is no reason to assume that, after CP-E are introduced, their spread will be less dynamic than that of ESBL-E. This inference has serious implications for the implementation of screening methods and effective infection control strategies. On the basis of the results of this study, we recommend active surveillance of CP-E and ESBL-E and at least temporary contact isolation precautions for patients being admitted to hospitals after travel to Asia during the previous 6 months.

Technical appendix Personal and travel characteristics of a cohort of 338 travelers from the Netherlands and risk factors for acquisition of extended-spectrum β-lactamase–producing *Enterobacteriaceae*.
