# Supplementary material for: Extended-Spectrum β-Lactamase–producing Enterobacteriaceae among Travelers from the Netherlands
Source: Emerg Infect Dis. 2013 Aug;19(8):1206–13. doi: 10.3201/eid.1908.130257 (PMC3739527; doi:10.3201/eid.1908.130257)
Supplement: Technical appendix — Personal and travel characteristics of a cohort of 338 travelers from the Netherlands and risk factors for acquisition of extended-spectrum β-lactamase–producing Enterobacteriaceae. [file 13-0257-Techapp-s1.pdf]

Article DOI: http://dx.doi.org/10.3201/eid1908.120257

Extended-Spectrum β-Lactamase–producing *Enterobacteriaceae* among Travelers from the Netherlands

Technical Appendix

Technical Appendix Table. Person and travel characteristics and risk factors for extended-spectrum β-lactamase producing *Enterobacteriaceae* acquisition in a cohort of 338 travelers from the Netherlands*

Variable	No. (%) negative pre- and post-travel, N = 225	No. (%) positive post-travel only, N = 113	Univariate analysis OR (95% CI)	p value†	Multivariate analysis OR (95% CI)	p value
Gender, female	144 (64.0)	69 (61.1)	0.88 (0.55–1.41)	0.60		
Age						
18–25 y	54 (24.0)	28 (24.8)	1.0			
26–33 y	66 (29.3)	24 (21.2)	0.39 (0.17–0.92)	0.03		
34–51 y	56 (24.9)	28 (24.8)	0.67 (0.28–1.61)	0.37		
≥52 y	43 (21.8)	33 (29.2)	1.46 (0.60–3.54)	0.41		
Vegetarian	13 (5.8)	6 (5.3)	0.91 (0.34–2.47)	0.86		
Health care worker	59 (26.2)	27 (23.9)	0.88 (0.52–1.49)	0.64		
Daily contact with farm animals	8 (3.6)	4 (3.5)	1.0 (0.29–3.38)	0.99		
Visit to identified risk areas during previous12 mo			1.01 (0.64–1.61)	0.96		
None	138 (61.3)	69 (61.1)				
Africa	26 (11.6)	15 (13.3)				
Asia	21 (9.3)	12 (10.6)				
India	5 (2.2)	4 (3.5)				
Middle East	26 (11.6)	13 (11.5)				
Central America and Carribean Region	17 (7.6)	7 (6.2)				
South America	6 (2.7)	5 (4.4)				
Medical problem ‡						
None	161 (71.6)	84 (74.3)				
Inflammatory bowel disease	2 (0.9)	1 (0.9)				
Chronic diarrhea	3 (1.3)	0				
Chronic constipation	3 (1.3)	1 (0.9)				
Irritable bowel syndrome	17 (3.1)	7 (6.2)				
Diabetes mellitus	3 (1.3)	1 (0.9)				
Gastroesophageal reflux	12 (5.3)	4 (3.5)				
Recurrent UTIs	4 (1.8)	1 (0.9)				
Autoimmune disease	7 (3.1)	2 (1.8)				
Abdominal pain of unknown origin	5 (2.2)	2 (1.8)				
Gallbladder problems	4 (1.7)	1 (0.9)				
Transplantation	1 (0.4)	0				
Celiac disease	0	2 (1.8)				
Other	30 (13.3)	18 (15.9)				
Antibiotic use during 12 mo before travel	47 (20.9)	17 (15.1)	0.85 (0.56–1.29)	0.45		
Hospitalization during 12 mo before travel						
<3 mo prior	5 (2.2)	3 (2.7)				
3–6 mo prior	2 (0.9)	1 (0.9)				
6–9 mo prior	1 (0.4)	1 (0.9)				
9–12 mo prior	2 (0.9)	2 (1.8)				
Travel destination, by subcontinents§						
Southeast Asia	73 (32.4)	37 (32.7)	1.01 (0.63–1.64)	0.96		

Variable	No. (%) negative pre- and post-travel, N = 225	No. (%) positive post-travel only, N = 113	Univariate analysis OR (95% CI)	p value†	Multivariate analysis OR (95% CI)	p value
East Asia	11 (4.9)	22 (19.5)	4.70 (2.19–10.1)	<0.001	3.95 (1.78–8.73)	0.001
South Asia	7 (3.1)	18 (15.9)	5.90 (2.39–14.60)	<0.001	5.09 (2–12.92)	0.001
Central Asia	2 (0.9)	1 (0.9)	1.0 (0.089–1.11)	0.99		
Middle East	13 (5.8)	2 (1.8)	0.29 (0.07–1.33)	0.11	0.28 (0.06–1.30)	0.103
North Africa	6 (2.7)	4 (3.5)				
Central Africa	39 (17.3)	17 (15.0)				
Southern Africa	23 (10.2)	3 (2.7)	0.24 (0.07–0.82)	0.02	0.24 (0.07–0.85)	0.027
Central America and the Caribbean	21 (9.3)	7 (6.2)	0.64 (0.26–1.56)	0.33		
South America	30 (13.3)	2 (1.8)	0.12 (0.027–0.50)	0.004	0.14 (0.03–0.59)	0.008
Median duration of stay, d (range)	21 (6–90)	22 (6–89)	0.99 (0.976–1.004)	0.17	1.0 (0.97–1.0)	0.22
Type of travel						
Self-arranged travel	95 (42.2)	52 (46.0)	1.17 (0.74–1.84)	0.51		
Backpacking	51 (22.7)	25 (22.1)	0.97 (0.56–1.67)	0.91		
Organized group travel	62 (27.6)	27 (23.9)	0.83 (0.49–1.39)	0.47		
Cruise	1 (0.4)	0				
Other	16 (7.1)	9 (8.0)				
Own (holiday) home	16 (7.1)	3 (2.7)				
Other	3 (1.3)	3 (2.7)				
Reason for travel						
Vacation	166 (73.8)	83 (73.5)				
Visit family/friends	8 (3.6)	8 (7.1)				
Business	15 (6.7)	9 (8.0)				
Study	18 (8.0)	7 (6.2)				
Volunteer work	10 (4.4)	5 (4.4)				
Travel group composition						
Alone	25 (11.1)	14 (12.4)	1.13 (0.56–2.27)	0.73		
With 1 partner	102 (45.3)	44 (38.9)	0.77 (0.49–1.22)	0.26		
More partners	44 (19.6)	30 (26.5)				
Group travel	54 (24.0)	25 (22.1)	1.23 (0.78–1.93)	0.37		
Accommodation during travel						
Luxury hotels	78 (34.7)	34 (30.1)	0.81 (0.50–1.32)	0.40		
Hostels	50 (22.2)	30 (26.5)	1.27 (0.75–2.13)	0.38		
Budget hotels	49 (21.8)	27 (23.9)	1.13 (0.66–1.93)	0.66		
Own holiday home	16 (7.1)	3 (2.7)				
Camping	10 (4.4)	6 (5.3)				
With family/friends	8 (3.6)	5 (4.4)				
Locals	7 (3.1)	3 (2.7)				
Boat	4 (1.8)	2 (1.8)				
Other	3 (1.3)	3 (2.7)				
Diarrhea during travel	83 (36.9)	45 (39.8)	1.13 (0.71–1.80)	0.60		
Companion travelers with diarrhea	115 (51.1)	61 (54.0)	1.1 (0.71–1.77)	0.62		
Antibiotic use during travel	10 (4.4)	9 (18.0)	1.86 (0.73–4.72)	0.19	1.98 (0.72–5.47)	0.16

*Data are presented as no. (%), unless stated otherwise. Blank cells indicate no data available for value. OR, odds ratio; UTI, urinary tract infection.
†Variables with p < 0.2 in the univariate analysis were included in the multivariate logistic regression model.
‡Participants could report >1 medical problem.
§ Travel destinations visited by the travelers who completed the study were divided in 10 subcontinents (n = no. of travelers per destination. One participant could have visited >1 country): Southeast Asia: Cambodia (n = 21), Philippines (n = 1), Indonesia (n = 62), Laos (n = 9), Malaysia, (n = 27), Singapore (n = 9), Thailand (n = 30) and Vietnam (n = 17); East Asia: People's Republic of China (n = 39), Japan (n = 1), Mongolia (n = 4) and Taiwan (n = 1); South Asia: Bangladesh (n = 1), India, (n = 20) Maldives (n = 2), Nepal (n = 8) and Sri Lanka (n = 5); Central Asia: Kazakhstan,(n = 2), Kyrgyzstan (n = 2) Uzbekistan (n = 2) and Turkmenistan (n = 1); Middle East: Iran (n = 1), Jordan (n = 1), Turkey (n = 14) Emirates(n = 3); North Africa: Egypt (n = 10) and Morocco (n = 5); Central Africa: Benin (n = 1), Cameroon (n = 1), Congo (n = 7), Gambia (n = 2), Ghana (n = 1), Kenya (n = 30), Liberia (n = 1), Rwanda (n = 1), Sierra Leone (n = 1), Tanzania (n = 24) and Uganda (n = 9); Southern Africa: Angola (n = 1), Botswana (n = 5), Lesotho (n = 2), Madagascar (n = 3), Malawi (n = 5), Mauritius (n = 1), Mozambique (n = 2), Namibia (n = 7) South Africa (n = 19), Swaziland (n = 6), Zambia (n = 6)and Zimbabwe. (n = 1); Central America and the Caribbean: Belize (n = 2), Bonaire (n = 1), Costa Rica (n = 9), Cuba (n = 5), Curacao (n = 1), Dominican Republic (n = 4), Grenada (n = 1), Guatemala (n = 4), Honduras (n = 2), Mexico (n = 9), Nicaragua (n = 5) and Panama (n = 3); South-America: Argentina (n = 3), Bolivia (n = 2), Brazil (n = 5), Chile (n = 2), Ecuador (n = 3), Guyana (n = 3, Peru (n = 3), Surinam (n = 20), Trinidad and Tobago (n = 2) and Venezuela (n = 1).
